# Therapeutic Potential of Winged Bean (*Psophocarpus tetragonolobus*) Pod Extract: Apoptosis Induction and Anti-Inflammatory Action in Colorectal Cancer Cells

**DOI:** 10.3390/biology14121646

**Published:** 2025-11-22

**Authors:** Pongsathorn Dhumtanom, Anurak Wongta, Wasin Wongwilai, Siriporn Okonogi, Wantida Chaiyana

**Affiliations:** 1 Herbs and Functional Products Research Unit, Multidisciplinary Research Institute, Chiang Mai University, Chiang Mai 50200, Thailand; pongsathorn.d@cmu.ac.th; 2 School of Health Science Research, Research Institute for Health Sciences, Chiang Mai University, Chiang Mai 50200, Thailand; anurak.wongta@cmu.ac.th; 3 Renewable Energy and Energy Conservation Research Unit, Multidisciplinary Research Institute, Chiang Mai University, Chiang Mai 50200, Thailand; wasin.w@cmu.ac.th; 4 Department of Pharmaceutical Sciences, Faculty of Pharmacy, Chiang Mai University, Chiang Mai 50200, Thailand; siriporn.okonogi@cmu.ac.th; 5 Center of Excellence in Pharmaceutical Nanotechnology, Faculty of Pharmacy, Chiang Mai University, Chiang Mai 50200, Thailand; 6 Multidisciplinary and Interdisciplinary School, Chiang Mai University, Chiang Mai 50200, Thailand

**Keywords:** winged bean, *Psophocarpus tetragonolobus*, colorectal cancer, HT-29 cells, apoptosis induction, anti-inflammation

## Abstract

*Psophocarpus tetragonolobus* (L.) DC or winged bean is a nutrient-rich tropical legume that has been a part of the Southeast Asian cuisine for centuries. In this study, phytochemical composition and anticancer and anti-inflammatory properties of winged bean pod ethanolic extract (WBE) were investigated with specific emphasis on colorectal cancer. Phytochemical fingerprint of WBE was evaluated by high-performance liquid chromatography (HPLC). Cytotoxicity and induction of apoptosis were evaluated in human colorectal adenocarcinoma (HT-29) by 3-(4,5-Dimethylthiazol-2-yl)-2,5-diphenyltetrazolium bromide (MTT) assay, morphological observation, and deoxyribonucleic acid (DNA) fragmentation. Anti-inflammatory activity was measured in lipopolysaccharide-stimulated mouse mononuclear macrophage (RAW 264.7) by determination of pro-inflammatory cytokines. WBE contained high total phenolic (237.33 ± 8.48 mg gallic acid/g) and flavonoid (180.53 ± 6.31 mg quercatin/g) content, with the primary flavonoid being kaempferol (0.012 g%). WBE dose-dependently suppressed viability (IC_50_ = 117.86 µg/mL) and triggered apoptosis in HT-29 cells, with morphological alterations and DNA fragmentation. In addition, WBE significantly suppressed secretion of interleukin-1β, interleukin-6, and tumor necrosis factor-α. Therefore, WBE displayed therapeutic potential for the induction of apoptosis against colorectal cancer cells and inhibitions of inflammatory cytokines. Despite being traditionally consumed as an edible plant, additional studies are needed to confirm the safety of *P. tetragonolobus* extract.

## Simple Summary

Colorectal cancer is one of the major causes of cancer in the world, and greater emphasis has been placed on natural foods with the capacity to prevent or slow its onset. The winged bean is a local food crop commonly consumed in Southeast Asia, and it contains numerous natural plant components with established health effects. Through this research, we established whether winged bean pod extract can inhibit cancer and inflammation. We discovered that the extract contained extremely high concentrations of natural antioxidants, i.e., molecules that are known to protect cells against damage. When the extract was applied to human colorectal cancer cells, growth of cancer cells was suppressed and programmed cell death was initiated, a process where the abnormal cells or damaged cells undergo apoptosis. Moreover, when applied to immune cells, the extract suppressed production of molecules that induce inflammation. These findings indicate winged bean pods could produce health impacts beyond plain nutrition. With more research, they might be created as a functional food or natural supplement to the promotion of colorectal cancer prevention and general well-being.

## 1. Introduction

Colorectal cancer ranks among the most prevalent malignancies globally and is a major contributor to cancer-related morbidity and mortality, representing the third most frequently diagnosed cancer and the fourth leading cause of cancer deaths worldwide [1]. Colorectal cancer killed 930,000 people and caused 1.9 million new cases in the 2020s, and by 2040, the burden is predicted to increase to 3.2 million cases and 1.6 million fatalities, particularly in highly developed nations [2]. Recent hypotheses suggested that inflammation and damage to the colorectal mucosa from environmental factors may play a key role in the early stages of colorectal carcinogenesis [3]. Despite being the fundamental basis of treatment, conventional therapies such as surgery, chemotherapy, and radiotherapy are often connected to side effects, low specificity, and recurrence risk [4]. Finding natural substances from food sources that can support traditional treatments by focusing on several pathways involved in the development of cancer, especially by causing apoptosis in cancerous cells and regulating inflammatory reactions that aid in the growth of tumors, is therefore becoming more and more popular.

Promising options for colorectal cancer prevention and adjuvant treatment include functional foods and bioactive compounds derived from plants. Legumes are known for their high protein content, dietary fiber, vitamins, minerals, and secondary metabolites like lectins, flavonoids, and phenolic acids [5,6]. These substances have a variety of bioactivities, such as anti-inflammatory, anti-proliferative, and antioxidant properties, which are strongly linked to immunomodulation and cancer prevention [7]. Despite extensive research on commonly consumed legumes such as soybeans and chickpeas, other nutrient-rich tropical legumes remain underexplored in terms of their therapeutic potential.

Winged bean (*Psophocarpus tetragonolobus* (L.) DC) has attracted considerable interest due to its widespread consumption and nutritional importance in Southeast Asian diets. Winged bean is a self-pollinated tropical legume in the Fabaceae family that is widely cultivated in Southeast Asia, including Thailand, India, Myanmar, Sri Lanka, and the Philippines [8]. Winged bean is considered a multi-purpose crop with all plant parts being edible, typically eaten fresh, either as pods or tubers [9,10]. The seeds contain 29 to 37% protein and 15 to 18% oil [11], while tubers contain approximately 20% protein [12], along with significant amounts of vitamins A and C, calcium, and iron [11]. In traditional medicine, winged bean leaves and seeds have been used to manage inflammatory skin conditions such as boils, ulcers, and smallpox lesions, highlighting their ethnopharmacological significance [13,14]. In addition, several scientific studies have reported the antibacterial, anti-proliferative, and fungicidal properties of winged bean, along with its antioxidant anti-inflammatory and immunomodulating activities [15,16,17]. However, most existing research has focused on extracts from the roots or leaves. In contrast, studies on the biological activities of the pods, the most commonly consumed part, remain limited.

Therefore, the purpose of this study was to evaluate the phytochemical profile of ethanolic extracts from winged bean pods (WBE), determine their anti-inflammatory properties, and assess their ability to induce apoptosis in HT-29 human colorectal adenocarcinoma cells.

## 2. Materials and Methods

### 2.1. Chemicals and Reagents

Standard phenolic compounds, including kaempferol (≥98% purity, CAS No. 520-18-3, molecular formula C_15_H_10_O_6_, molecular weight 286.24 g/mol), quercetin (≥95% purity, CAS No. 117-39-5, molecular formula C_15_H_10_O_7_, molecular weight 302.24 g/mol), rutin (≥95% purity, CAS No. 153-18-4, molecular formula C_27_H_30_O_16_, molecular weight 610.52 g/mol), apigenin (≥98% purity, CAS No. 520-36-5, molecular formula C_15_H_10_O_5_, molecular weight 270.24 g/mol), and gallic acid (≥98% purity, CAS No. 149-91-7, molecular formula C_7_H_6_O_5_, molecular weight 170.12 g/mol)n, were purchased from Sigma-Aldrich (St. Louis, MO, USA). Tannic acid (CAS No. 1401-55-4, molecular formula C_76_H_52_O_46_, molecular weight 1,701.20 g/mol) was purchased from BMG Labtech GmbH (Ortenberg, Germany). Isoquercitrin (≥98% purity, CAS No. 482-35-9; molecular formula C_21_H_20_O_12_; molecular weight 464.38 g/mol), also known as quercetin 3-O-β-D-glucoside, was purchased from Cayman Chemical (Ann Arbor, MI, USA). Folin–Ciocalteu’s phenol reagent was obtained from Merck & Co., Inc (Kenilworth, NJ, USA). Ammonium chloride, 2,2-diphenyl-1-picrylhydrazyl (DPPH), dimethyl sulfoxide (DMSO), 3-(4,5-dimethylthiazol-2-yl)-2,5-diphenyltetrazolium bromide (MTT), 2′,7′-dichlorofluorescin diacetate, 6-hydroxy-2,5,7,8-tetramethylchroman-2-carboxylic acid (Trolox), tocopherol (TC), phosphate-buffered saline (PBS), and thiobarbituric acid, were also supplied from Sigma-Aldrich (St. Louis, MO, USA). Dulbecco’s modified eagle medium (DMEM) was purchased from Gibco (Thermo Fisher Scientific, Waltham, MA, USA). Fetal bovine serum (FBS), penicillin–streptomycin, and trypsin– ethylenediaminetetraacetic acid (EDTA) were obtained from Hyclone (Logan, UT, USA). Proteinase K, K-buffer, and RNase A were purchased from Vivantis Technologies Sdn. Bhd. (Subang Jaya, Selangor, Malaysia). Human colon adenocarcinoma (HT-29) and murine macrophage (RAW 264.7) cell lines were obtained from ATCC (Manassas, VA, USA). The DNA ladder (100 bp, H3 RTU) was obtained from GeneDireX, Inc., (Las Vegas, NV, USA).

### 2.2. Plant Material and Extraction

Mature winged bean pods were purchased from local markets in Chiang Mai, Thailand. The pods were washed, chopped, and dried at 50 °C in a hot air oven (Memmert GmbH & Co. KG, Schwabach, Germany), then ground into a fine powder using a mechanical grinder (Remi, Hyderabad, India). The dried powder was macerated overnight at room temperature in 70% ethanol (1:10 *w*/*v*). After filtration, the extract was concentrated and lyophilized using a freeze dryer (Beta 2–8 LD-plus, Martin Christ Gefriertrocknungsanlagen GmbH, Osterode am Harz, Germany) to obtain the WBE. All extracts were stored at −20 °C until use. The extraction yield was calculated using the following equation:

Extract yield (%) = A/B × 100,(1)

where A represents dry weight (g) of the extract and B represents dry weight (g) of the plant materials.

### 2.3. Cell Lines and Culture Conditions

HT-29 and RAW 264.7 cell lines were cultured in DMEM supplemented with 10% FBS, 100 U/mL penicillin, and 100 U/mL streptomycin. Cells were maintained at 37 °C in a humidified atmosphere with 5% CO_2_.

### 2.4. Determination of Chemical Compositions of WBE

#### 2.4.1. Determination of Total Phenolic Content Using Folin–Ciocalteu Method

Total phenolic content was determined using the Folin–Ciocalteu method [18] and expressed as milligrams of gallic acid equivalent per gram of extract (mg GAE/g extract). Briefly, 100 μL of the sample solution (1 mg/mL in methanol) was mixed with 2.9 mL of DI water, 0.5 mL of Folin–Ciocalteu reagent, and 2.0 mL of 20% Na_2_CO_3_ solution. The mixture was left to stand for 90 min at room temperature, and absorbance was measured at 760 nm using a UV-Vis spectrophotometer (model 8453, Agilent Technologies, Santa Clara, CA, USA). A calibration curve was prepared using GA standard solutions (10–100 μg/mL) and used to calculate the total phenolic content. All experiments were performed in triplicate.

#### 2.4.2. Determination of Total Flavonoid Content Using Aluminum Chloride Method

Total flavonoid content was measured using an aluminum chloride colorimetric method adapted from Elin Novia et al. (2018) [19], with minor modifications. Q was used as the standard. In a 96-well plate, 50 μL of 1 mg/mL extract or standard solution was mixed with 10 μL of 10% aluminum chloride solution and 150 μL of 96% ethanol. Then, 10 μL of 1 M sodium acetate was added. The plate was kept protected from light and incubated at room temperature for 40 min. Absorbance was measured at 415 nm using a SPECTROStar Nano microplate reader ((SPECTROStar Nano, BMG LABTECH, Ortenberg, Germany). Results were expressed as milligrams of quercetin equivalent per gram of extract (mg QE/g extract). All experiments were performed in triplicate.

#### 2.4.3. High-Performance Liquid Chromatography with Diode Array Detection (HPLC–DAD)

HPLC system (Agilent 1100, Agilent Technologies, Santa Clara, CA, USA) was used to determine the phenolic compound content of WBE, following the method described by Dhumtanom et al. (2025) [20] with minor modifications. Filtered samples were separated on a LiChroCART RP-18e column (4.6 × 150 mm, 5 μm particle size; Merck & Co., Inc., Kenilworth, NJ, USA). The mobile phases consisted of acetonitrile (phase A) and 10 mM ammonium formate buffer pH 4.0 (phase B). The gradient elution program was set as follows: 0–5 min, 100% phase B; 5–10 min, 0–20% phase A; 10–20 min, 20% phase A; and 20–60 min, 20–40% phase A. A diode array detector (Agilent Technologies, Santa Clara, CA, USA) was used to monitor phenolic peaks at 270, 330, 350, and 370 nm. Various concentrations of standard compounds, including gallic acid, tannic acid, quercetin, rutin, isoquercitrin, apigenin, and kaempferol, were prepared to construct calibration curves for the quantitative determination of each phenolic compound. All experiments were performed in triplicate.

### 2.5. Determination of Cytotoxic Effects Through Cell Viability and Proliferation Assays

The MTT assay described by Skehan et al. (1990) [21] was used to evaluate the cytotoxic effect of WBE on cell viability. HT-29 cells (10^4^ cells/well) were seeded in 96-well plates and treated with WBE at concentrations ranging from 10 to 300 μg/mL for 24 h. After incubation, 20 μL of MTT solution (5 mg/mL) was added to each well, and the plates were incubated at 37 °C for an additional 4 h. The resulting formazan crystals were dissolved in 200 μL of acidic isopropanol. Absorbance was measured at 570 nm using a SPECTROStar Nano microplate reader (BMG LABTECH, Ortenberg, Germany). Cell viability was calculated by comparing absorbance values to those of the untreated control. The percentage of growth inhibition was calculated using the following formula:

Cell growth inhibition (%) = 100 × (1 − A/B),(2)

where A represents the absorbance of the system with the sample and B represents the absorbance of the system without the sample. All experiments were performed in triplicate.

### 2.6. Determination of Apoptosis Induction

#### 2.6.1. Apoptotic Morphological Analysis

Morphological changes in HT-29 cells were observed using the acridine orange/ethidium bromide (AO/EB) staining method as described by Liu et al. (2015) [22] under a phase-contrast microscope (CKX-41, Olympus, Tokyo, Japan). In brief, following treatment with WBE at concentrations of 50–200 μg/mL for 24, 48, or 72 h, the cellular alterations were photographed to document apoptotic characteristics. Cells were incubated with a dual fluorescent solution containing 10 μg/mL each of AO and EB for 15 min in the dark. Stained cells were examined under a fluorescence microscope (CKX-41, Olympus Corporation, Tokyo, Japan). All experiments were performed in triplicate.

#### 2.6.2. DNA Fragmentation Analysis

DNA fragmentation, a biochemical hallmark of apoptosis, was analyzed to confirm the apoptotic process induced by WBE. The protocol was adapted from Ha et al. [23], with modifications. HT-29 cells were seeded at a density of 1 × 10^6^ cells/mL in 6-well plates. Upon reaching approximately 80% confluence, cells were treated with WBE at concentrations of 50–200 μg/mL for 24, 48, or 72 h. After treatment, the culture medium was removed, and cells were rinsed with PBS (137 mM NaCl, 2.7 mM KCl, 10 mM Na_2_HPO_4_, 1.8 mM KH_2_PO_4_). A total of 200 μL of lysis buffer (20 mM Tris-HCl, pH 7.5; 150 mM NaCl; 1 mM Na_2_EDTA; 1% Triton X-100) was added to each well, mixed thoroughly, and transferred to 1.5 mL microcentrifuge tubes. Subsequently, 20 μL of Proteinase K (5 mg/mL) and K-buffer were added, followed by incubation at 56 °C for 3 h on a shaking hot plate. To inactivate proteinase K, samples were heated to 95 °C for 10 min. Next, 200 μL of RNase A buffer and 20 μL of RNase A (5 mg/mL) were added and further incubated at 37 °C for 1 h. DNA was precipitated by adding 10 μL of 3 M sodium acetate and two volumes of ice-cold isopropanol, followed by overnight incubation at −20 °C. Samples were then centrifuged at 14,000 rpm for 30 min at 4 °C. The supernatant was discarded, and pellets were air-dried for at least 2 h before being resuspended in 200 μL of Tris(hydroxymethyl)aminomethane–ethylenediaminetetraacetic acid (TE) buffer. DNA samples were stored at −20 °C until electrophoresis. Electrophoresis was carried out on 1.5% agarose gels containing ethidium bromide, and DNA bands were visualized to assess fragmentation. All experiments were performed in triplicate.

### 2.7. Determination of Anti-Inflammatory Effects

To evaluate the anti-inflammatory potential of WBE, the production of key pro-inflammatory cytokines, including interleukin-6 (IL-6), interleukin-1β (IL-1β), and tumor necrosis factor-alpha (TNF-α), was measured in LPS-stimulated RAW 264.7 macrophages. In 6-well plates, 5 × 10^5^ cells/well of RAW 264.7 cells were cultured in DMEM with 1 µg/mL of LPS for 24 h. Thereafter, cells were rinsed twice with PBS and continuously cultured with various concentrations of WBE for 24 h. Finally, a cell culture supernatant was subjected to an evaluation of the concentration of IL-6, IL-1β, and TNF-α by ELISA kit (Abcam, Cambridge, UK) according to the manufacturer’s instructions. All experiments were performed in triplicate.

### 2.8. Statistical Analysis

All experimental data were expressed as mean ± standard deviation (SD). Statistical analysis was performed using one-way analysis of variance (ANOVA), followed by Tukey’s post hoc test for multiple comparisons, using GraphPad Prism version 9.0.0 (GraphPad Software, San Diego, CA, USA). Differences were considered statistically significant at *p* < 0.05.

## 3. Results

### 3.1. Phytochemical Composition of WBE

**Figure 1:**
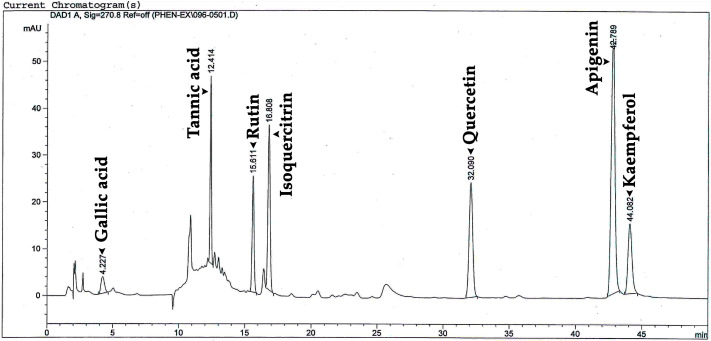
HPLC chromatogram at 270 nm of WBE, showing major phenolic compounds, including gallic acid, tannic acid, rutin, isoquercitrin, quercetin, apigenin, and kaempferol.

The extraction yield of WBE was 7.34 ± 0.65% *w*/*w*. The phytochemical composition of WBE, as evaluated by HPLC, is presented in Figure 1, and the content of each phenolic component is listed in Table 1. The HPLC chromatogram of WBE revealed the presence of several phenolic and flavonoid compounds. Major peaks were identified by comparison with authentic standards, corresponding to gallic acid (2.27 min), tannic acid (9.14 min), rutin (15.81 min), isoquercitrin (18.68 min), quercetin (32.00 min), apigenin (42.78 min), and kaempferol (44.06 min). However, the gradient used in this study was less economical in terms of runtime. Therefore, further improvement of the chromatographic conditions was encouraged in future investigations, particularly through systematic optimization of gradient slope, column type, and mobile phase composition.

The phytochemical composition of the ethanolic extract from winged bean pods as summarized in Table 1 noted a high content of both phenolic and flavonoid compounds. It should be noted that the areas of the chromatographic peaks in Figure 1 do not directly correspond to the concentrations reported in Table 1. Quantification was performed using individual standard curves for each compound, which accounted for differences in detector response at the fixed detection wavelength of 270 nm. Although apigenin exhibited a very high peak, whereas kaempferol showed a relatively small peak at 270 nm, this apparent discrepancy arose from differences in their detector response and standard curves. In HPLC quantification, peak height or area alone did not directly reflect absolute concentration. The measured signal should be interpreted using compound-specific calibration curves, which are necessary to account for differences in detector response [24]. Apigenin and kaempferol have different molar absorptivity and response at 270 nm, resulting in standard curves with different slopes. Consequently, a small peak for kaempferol can correspond to a much higher concentration than a large peak for apigenin. This illustrated why quantitative determination should rely on standard curves rather than visual inspection of chromatograms, ensuring accurate measurement of each compound regardless of differences in UV absorbance or peak appearance.

Among the identified constituents, flavonols such as kaempferol, quercetin, and their derivatives were predominant, whereas phenolic acids including tannic and gallic acids were present in lower amounts. These findings indicate that WBE is particularly rich in flavonoid-based phytochemical constituents, that may contribute to its biological activities. However, it should be noted that the identification and relative abundance of these compounds are based on UV-detectable chromophores. Complex polyphenols, especially galloyl tannins, are structurally diverse and may not be fully represented under the current chromatographic conditions. Moreover, the use of single-compound standards provided a retention reference but might not capture the heterogeneity of naturally occurring tannins, whose purity and molecular distributions could vary across botanical sources [25]. Therefore, while the present profile reflects the major UV-responsive constituents, comprehensive characterization of non-UV chromophore tannins and other high-molecular-weight phenolics would require further method optimization, such as employing more acidic eluents and lower-ramped gradients, and potentially bioassay-guided fractionation to better define the active phytochemical contributors.

### 3.2. Cytotoxic Effects of WBE

**Figure 2:**
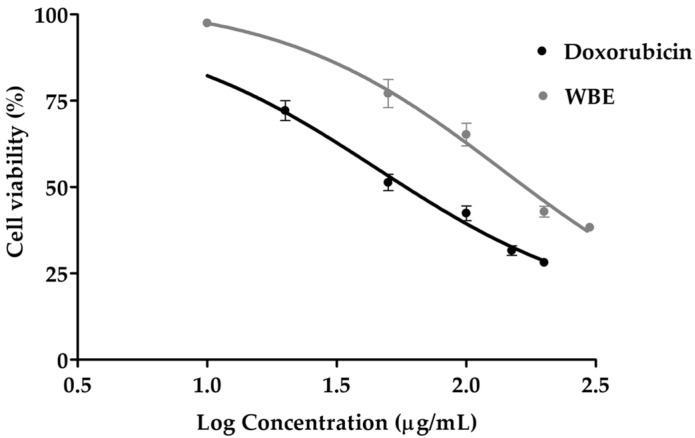
Dose–response curve of Doxorubicin and WBE on HT-29 cells viability.

The cytotoxic effects of doxorubicin and WBE on HT-29 human colon cancer cells were evaluated using the MTT assay after 24 h of exposure to concentrations ranging from 10 to 300 μg/mL. This concentration range was selected based on preliminary dose–response experiments to ensure that both sub-toxic and cytotoxic effects could be captured, allowing accurate assessment of cell viability, reliable calculation of IC_50_ values, and comparison of the dose-dependent effects of the plant extract relative to the standard chemotherapeutic agent. Moreover, the selected range is scientifically justified when compared with previously reported cytotoxic concentrations of doxorubicin, which range from 0.05 to 10 mM (equivalent to 27.18–5435.2 µg/mL), confirming that the tested doses fall within an experimentally relevant window [26]. Both treatments demonstrated a significant dose-dependent reduction in cell viability, as shown in Figure 2. At lower concentrations (≤10 μg/mL), WBE exhibited minimal cytotoxicity, maintaining cell viability above 80%. In contrast, higher concentrations of WBE (≥50 μg/mL) significantly reduced cell viability (*p* ≤ 0.002), with the lowest values observed at 200 and 300 μg/mL (42.82 ± 4.43% and 38.30 ± 2.81%, respectively). Doxorubicin displayed stronger cytotoxicity, with an IC_50_ of 45.8 ± 9.4 μg/mL, compared to 105.3 ± 5.4 μg/mL for WBE, highlighting WBE’s moderate but notable cytotoxic activity against HT-29 cells. The lower cytotoxicity of WBE compared to doxorubicin at equivalent concentrations may be attributed to several potential mechanisms. Unlike doxorubicin, which is a potent chemotherapeutic agent that inhibits topoisomerase IIα, causing DNA strand breaks, disruption of replication, and apoptosis [27], WBE is a complex natural extract containing multiple bioactive compounds that may act more subtly. This difference is likely due to the fundamental distinctions between pure compounds and natural extracts. WBE contains a mixture of bioactive constituents with diverse chemical structures, and interactions among these compounds may lead to synergistic, additive, or even antagonistic effects, often resulting in more moderate modulation of cellular pathways [28,29]. Consequently, while doxorubicin induced strong ROS-mediated apoptosis in cancer cells [27], WBE, enriched with antioxidant compounds [30], counteracted oxidative stress and helped preserve cell viability, which likely accounted for its lower cytotoxicity. Given that HT-29, a well-established human colon adenocarcinoma cell line, is commonly used to screen potential chemotherapeutic agents and investigate colorectal cancer mechanisms [31], these results suggested that WBE could be a promising anticancer candidate based on its demonstrated cytotoxic effects.

### 3.3. Apoptosis Induction of WBE

**Figure 3:**
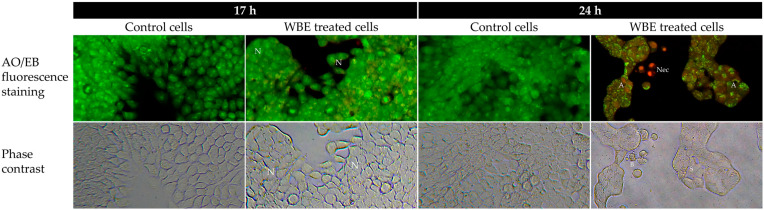
Acridine orange/ethidium bromide (AO/EB) fluorescence micrographs (**top**) and phase-contrast images (**bottom**) of HT-29 cells after treatment with 100 µg/mL WBE for 17 h and 24 h compared with untreated control cells. Normal morphology and intact green fluorescence indicate viable cells, while orange to red fluorescence represents necrotic or late apoptotic cells. Images were captured using a fluorescence microscope (CKX-41, Olympus Corporation, Tokyo, Japan) at a magnification of 400×. N represents normal cells; A, apoptotic bodies; Nec, necrotic cells; S, cell shrinkage; and B, membrane blebbing.

**Figure 4:**
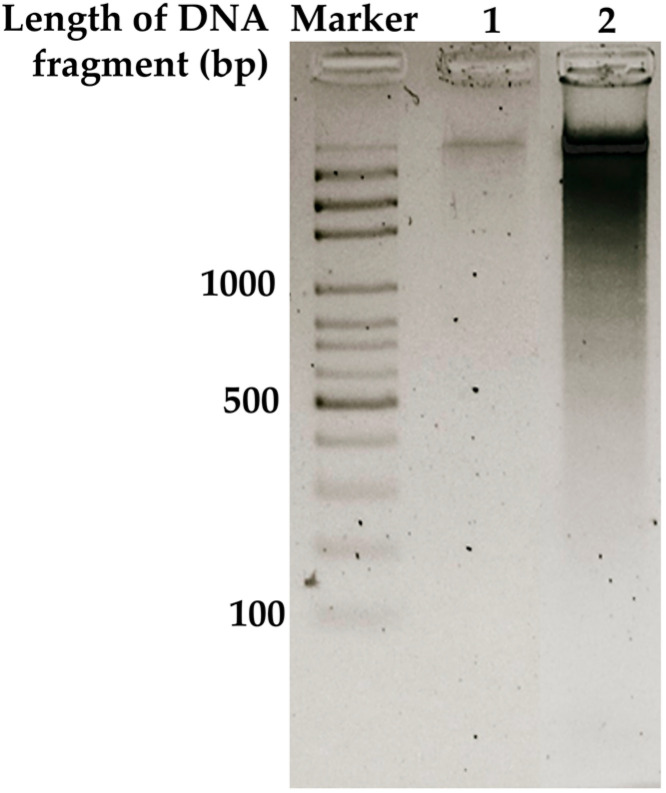
DNA fragmentation of HT-29 cells treated with WBE. Lane 1: untreated control cells; Lane 2: HT-29 cells treated with 100 µg/mL WBE for 24 h; Marker: 100 bp DNA ladder.

WBE-treated HT-29 cells were evaluated for apoptotic morphological changes and DNA fragmentation, and the findings are shown in Figure 3 and Figure 4, respectively. Apoptotic morphological changes were observed through AO/EB staining, a technique used to differentiate live, apoptotic, and necrotic cells based on membrane integrity and nuclear morphology [32]. Live cells were stained only with AO, exhibiting green fluorescence, whereas apoptotic cells were stained with both AO and EB, as EB can enter cells with compromised membranes [33,34]. Necrotic cells appeared orange to red due to EB staining and displayed less distinct nuclear morphology compared to viable cells [33,34].

Treatment of HT-29 cells with 100 µg/mL WBE induced noticeable cytotoxic effects in a time-dependent manner, as observed by AO/EB fluorescence and phase-contrast microscopy as shown in Figure 3. Untreated control cells exhibited normal morphology with intact green fluorescence, indicating high viability. After 17 h of WBE exposure, a subset of cells displayed orange fluorescence along with early apoptotic features such as cell shrinkage and membrane blebbing. By 24 h, the proportion of cells showing orange to red fluorescence increased markedly, accompanied by more pronounced morphological changes, including shrinkage, blebbing, and signs of necrosis. These findings suggested that WBE treatment promotes both apoptosis and necrosis in HT-29 cells over time. Apoptosis is a tightly regulated, signal-dependent form of cell death, whereas necrosis is a distinguishable, uncontrolled response to severe stress, characterized by membrane disruption, organelle swelling, DNA degradation, and inflammation [35,36]. However, apoptosis and necrosis may occur sequentially within the same cell or concurrently in distinct cells within the population [36,37]. A subset of cells might initially undergo apoptosis, but prolonged exposure or cumulative cellular stress shifted the death pathway toward necrosis [37], resulting in the simultaneous observation of both apoptotic and necrotic features at 24 h. The likely explanation for the transition from apoptosis to necrosis is that HT-29 cells are prone to necrosis under certain stress conditions, particularly when apoptosis is compromised due to p53 mutations or alterations in Bcl-2 family protein regulation [37].

In addition, DNA fragmentation analysis was performed to investigate the apoptotic effect of WBE on HT-29 cells. As shown in Figure 4, untreated control cells (Lane 1) displayed intact, high-molecular-weight DNA, indicating no apoptosis. In contrast, HT-29 cells treated with 100 µg/mL WBE for 24 h (Lane 2) exhibited a pronounced DNA smear extending from high- to low-molecular-weight fragments, consistent with extensive DNA cleavage associated with apoptosis. The 100 bp DNA ladder (Marker) was used as a size reference. These results confirm that WBE induces DNA fragmentation in HT-29 cells, supporting its pro-apoptotic activity.

### 3.4. Anti-Inflammatory Effects of WBE

The anti-inflammatory effects of WBE on LPS-stimulated RAW 264.7 macrophages were evaluated by assessing its inhibitory effects on IL-1β, IL-6, and TNF-α secretion. These cytokines are key pro-inflammatory mediators implicated in the pathogenesis and progression of colorectal cancer, where chronic inflammation promotes tumor development and drives cell proliferation, angiogenesis, and metastasis [38]. Additionally, clinical data showed that plasma levels of IL-1β, IL-6, and TNF-α are significantly elevated in patients with colorectal cancer compared with healthy controls [38]. Therefore, inhibition of IL-1β, IL-6, and TNF-α may help reduce inflammation associated with colorectal cancer, potentially slowing tumor growth, angiogenesis, and metastasis.

**Figure 5:**
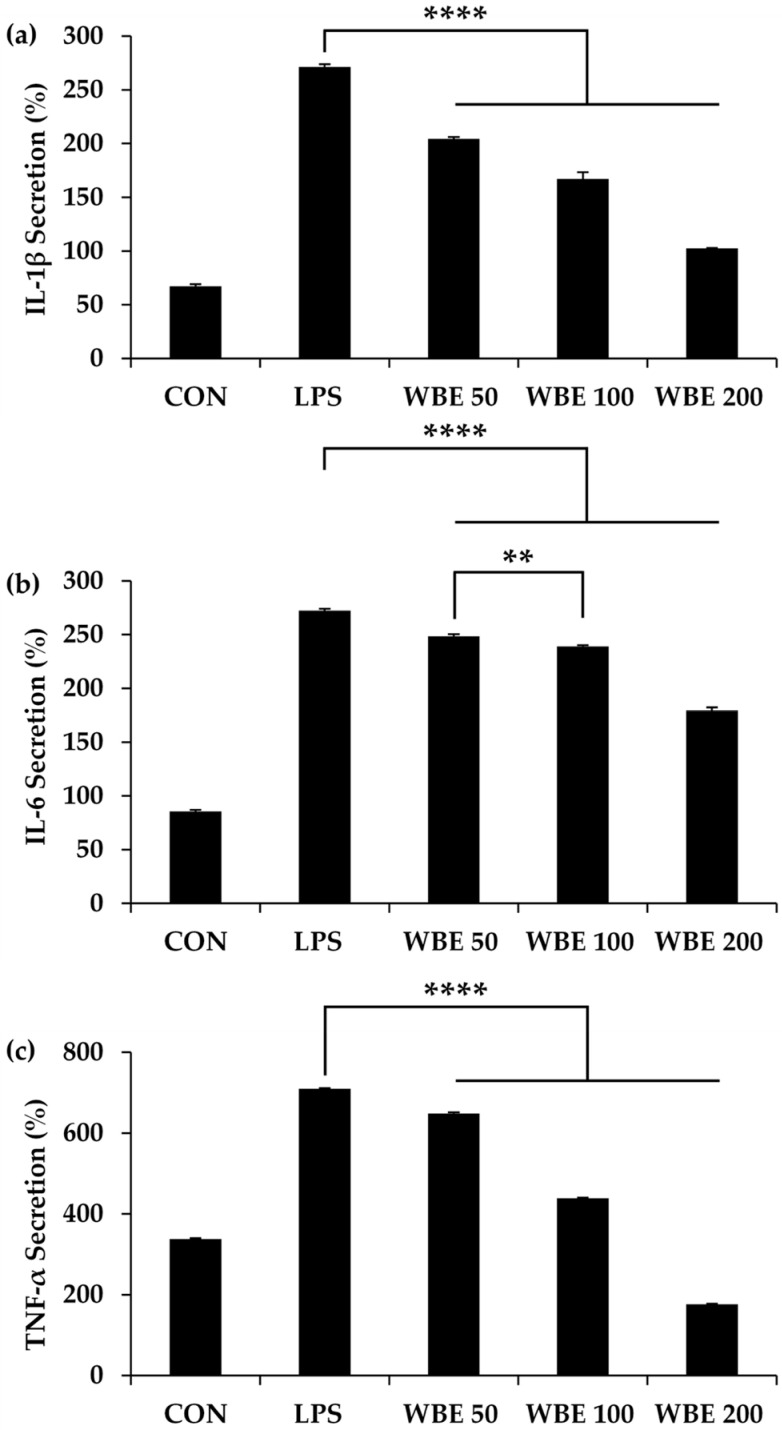
Effects of WBE on LPS-induced pro-inflammatory cytokine production in RAW 264.7 macrophages. Cells were stimulated with LPS (1 µg/mL) alone or co-treated with different concentrations of WBE (50, 100, or 200 µg/mL) for 24 h. The production levels of IL-1β (**a**), IL-6 (**b**), and TNF-α (**c**) were measured and expressed as percentages relative to the control. Data are presented as mean ± SD (*n* = 3). Statistical analysis was performed using one-way ANOVA followed by post hoc tests. Asterisks denote significant differences among groups (** *p* < 0.01, **** *p* < 0.0001 versus LPS-treated group).

To assess the anti-inflammatory effects of WBE, RAW 264.7 macrophages were stimulated with LPS for 24 h in the presence or absence of WBE. As shown in Figure 5, LPS stimulation significantly increased the secretion of IL-1β, IL-6, and TNF-α compared with untreated controls, confirming successful induction of an inflammatory response. Cytokine levels rose from 67.1 ± 1.5 pg/mL to 271.3 ± 1.5 pg/mL for IL-1β, from 85.6 ± 1.1 pg/mL to 272.2 ± 1.5 pg/mL for IL-6, and from 338.0 ± 1.6 pg/mL to 710.2 ± 0.9 pg/mL for TNF-α (*p* < 0.0001). Interestingly, the treatment with WBE significantly and dose-dependently attenuated LPS-induced cytokine production. At 200 µg/mL, WBE reduced IL-1β, IL-6, and TNF-α levels to 102.4 ± 0.2 pg/mL, 179.1 ± 2.5 pg/mL, and 176.5 ± 1.2 pg/mL, respectively (*p* < 0.0001). Lower concentrations of WBE (50 and 100 µg/mL) also significantly suppressed IL-6 secretion (*p* < 0.0001), with a notable difference between the two doses (*p* < 0.01), although IL-1β and TNF-α were not significantly reduced at these concentrations. Therefore, these results indicate that WBE effectively mitigates LPS-induced inflammatory cytokine production in RAW 264.7 macrophages, particularly at higher concentrations. The observed dose-dependent reduction in IL-1β, IL-6, and TNF-α secretion may be associated with the inhibition of key inflammatory signaling pathways, including nuclear Factor κB (NF-κB) and mitogen-activated protein kinase (MAPK) [39,40,41,42]. NF-κB is a pivotal pro-inflammatory transcription factor that regulates the expression of multiple cytokines, growth factors, and genes involved in apoptosis, proliferation, and angiogenesis, and its aberrant activation is implicated in various inflammatory disorders and tumorigenesis [39,40]. Similarly, MAPK signaling cascades, including extracellular signal–regulated kinase (ERK), c-Jun N-terminal kinase (JNK), p38 mitogen-activated protein kinase (p38 MAPK), mediate inflammatory responses by activating transcription factors such as activator protein-1 (AP-1), which drive cytokine gene expression [41,42]. By modulating these pathways, WBE may attenuate LPS-induced activation of NF-κB and MAPK, thereby suppressing downstream cytokine production and providing a mechanistic basis for its potent anti-inflammatory effects. Specifically, similar studies have shown that catalposide suppressed nuclear translocation of the NF-κB p65 subunit and reduced LPS binding to CD14, thereby limiting TNF-α, IL-1β, and IL-6 expression [43]. Likewise, ascofuranone selectively inhibited ERK phosphorylation, decreased NF-κB and AP-1 nuclear translocation, and downregulated cytokine mRNA and protein levels in a dose-dependent manner [44].

Although these in vitro findings provide valuable mechanistic insights into the anti-inflammatory properties of WBE, it is important to recognize that in vivo efficacy may be influenced by other factors, such as bioavailability, metabolism, and tissue distribution of the bioactive compounds of WBE. Moreover, the complex interactions within the immune system and the tumor microenvironment could modulate inflammatory responses differently than observed in isolated cells [45,46]. Consequently, further in vivo studies are necessary to validate the anti-inflammatory potential of WBE, optimize dosing strategies, and assess its impact on colorectal cancer progression.

## 4. Discussion

The present study highlighted the therapeutic potential of WBE in colorectal cancer, demonstrating its pronounced anti-inflammatory and anti-cancer activities. The anti-cancer effects of WBE were substantiated by evidence of apoptosis, as confirmed through both cellular morphological assessments and DNA fragmentation analyses. According to phytochemical analysis, WBE is high in phenolic acids like gallic and tannic acid as well as flavonoids, especially kaempferol and quercetin. In line with earlier findings on the anti-inflammatory and anti-proliferative properties of flavonoids derived from plants, the preponderance of flavonols implies that these substances might be involved in the biological activities that have been observed [47,48]. It is well known that flavonoids, particularly kaempferol, cause cancer cells to undergo both intrinsic and extrinsic apoptosis [49].

Kaempferol, a naturally occurring flavonoid, has demonstrated anticancer activity across multiple cancer types, including breast (MDA-MB-453), ovarian (A2780/CP70, A2780wt, OVCAR-3), leukemia (HL-60, NB-4), liver (HepG2), glioblastoma (A172), and cervical (HeLa) cells [49]. Its anticancer effects were primarily mediated through apoptosis induction, involving mitochondrial pathways, upregulation of pro-apoptotic proteins and downregulation of anti-apoptotic proteins [50,51,52,53]. Kaempferol also inhibited proliferative signaling via the Protein Kinase B (Akt) and cellular myelocytomatosis oncogene (c-Myc) pathways, along with prevented angiogenesis through suppression of vascular endothelial growth factor [51,54]. Additionally, it can induce endoplasmic reticulum apoptosis [55] and enhance the effects of chemotherapeutic agents such as cisplatin [54]. These multi-targeted mechanisms suggested that kaempferol could have therapeutic potential in colorectal cancer, where dysregulated apoptosis, proliferation, and angiogenesis were key contributors to tumor progression. As kaempferol was identified as the major phytochemical constituent in WBE in the present study, it was likely a key contributor to the anti-cancer effects. HT-29 cells treated with WBE exhibited classical apoptotic features, including membrane blebbing, DNA fragmentation, and chromatin condensation. Nevertheless, other phenolic and flavonoid compounds present in the extract may also contribute to its therapeutic effects [56], and potential synergistic interactions among these constituents could play an important role in enhancing overall efficacy.

In addition to its anticancer properties, the present study demonstrated that WBE exerted potent anti-inflammatory activity by significantly reducing the secretion of key pro-inflammatory cytokines IL-1β, IL-6, and TNF-α in LPS-stimulated RAW 264.7 macrophages. Chronic inflammation has been known to play a role in the development and spread of colorectal cancer by encouraging the initiation, growth, angiogenesis, and metastasis of tumors [3,57]. Patients with colorectal cancer often have elevated levels of IL-1β, IL-6, and TNF-α, which are associated with aggressive tumor behavior and a poor prognosis [58]. WBE, capable of inhibiting the secretion of IL-1β, IL-6, and TNF-α in LPS-stimulated RAW 264.7 macrophages, may contribute to the attenuation of inflammation in colorectal cancer. Its rich phytochemical composition was largely responsible for its anti-inflammatory properties. The primary flavonoid in WBE, kaempferol, has been shown to inhibit NF-κB and STAT3 signaling, two key pathways that control the transcription of IL-1β, IL-6, and TNF-α [59,60]. Together with kaempferol, other phenolics in the extract may further inhibit inflammatory signaling and cytokine production [61]. These findings suggested that WBE not only exerted direct cytotoxic effects on cancer cells through apoptosis but also modulated the tumor microenvironment by dampening chronic inflammation, highlighting its dual therapeutic potential. Furthermore, these processes acted synergistically, with the direct induction of apoptosis ensuring the elimination of cancerous cells, while the suppression of inflammatory cytokines limited tumor cell survival and proliferation. This integrated approach aligned with current strategies in colorectal cancer therapy, where agents that target both tumor cells and the inflammatory microenvironment are considered more effective [62,63].

*P. tetragonolobus* has a long history of safe consumption, with all plant parts edible and commonly used in traditional dishes. The plant is nutritionally rich, containing high levels of protein, vitamins, and minerals [13]. Although anti-nutrients such as tannins, phytates, and trypsin inhibitors are present, typical dietary intake is unlikely to cause harm, and heat treatment effectively deactivates these compounds [64]. Therefore, the cytotoxicity observed in concentrated ethanolic extracts does not reflect the safety of consuming the whole plant as food, as traditional use involves fresh or cooked material rather than concentrated extracts. Phytochemical analysis of WBE identified kaempferol as the major component, along with tannic acid, quercetin, and other minor flavonoids and phenolics, which collectively demonstrated pro-apoptotic and anti-inflammatory effects in colorectal cancer models. While bioassay-guided fractionation represented the gold standard for precisely identifying active compounds, including those lacking chromophores that may escape detection through conventional phytochemical profiling, the present study primarily aimed to provide an initial characterization of WBE and to associate its major constituents with observed bioactivities. Further bioassay-guided fractionation would be necessary to comprehensively identify all active molecules and clarify their individual contributions to the pro-apoptotic and anti-inflammatory effects. These findings, combined with its established safety and nutritional profile, suggested that WBE is a promising, safe natural candidate for further development as an anti-cancer dietary supplement or therapeutic agent. However, additional in vivo studies are needed to confirm these results and clarify the mechanisms involved.

## 5. Conclusions

The current study highlighted the therapeutic potential of WBE against colorectal cancer by presenting strong evidence of its anti-inflammatory and anti-cancer effects. According to phytochemical profiling, WBE was abundant in phenolic acids like gallic and tannic acids as well as flavonoids, especially kaempferol and quercetin. However, this analysis relied on UV detection, which may not reveal compounds lacking chromophores. Therefore, further studies using bioassay-guided fractionation or more comprehensive analytical techniques would be warranted to fully characterize its phytochemical profile. Significant cytotoxicity was shown by WBE against HT-29 colon cancer cells, causing apoptosis as shown by DNA fragmentation, membrane blebbing, and morphological changes. Additionally, in LPS-stimulated RAW 264.7 macrophages, WBE successfully inhibited the production of important pro-inflammatory cytokines (IL-1β, IL-6, and TNF-α). Therefore, WBE would be a promising multi-targeted natural agent for the treatment of colorectal cancer due to its dual mechanism of action, which involves both the direct induction of apoptosis in cancer cells and the attenuation of inflammatory mediators. However, further mechanistic studies are recommended to confirm the dual anti-cancer and anti-inflammatory effects of WBE, including apoptosis pathway analysis in colorectal cancer cells, investigation of NF-κB and MAPK signaling in macrophages, and complementary in vivo experiments. Additionally, studies combining WBE with conventional chemotherapeutic agents are suggested to enhance its therapeutic efficacy while simultaneously reducing potential side effects.

## Figures and Tables

**Table 1:** Phytochemical composition of WBE.

Chemical Compositions	Concentration
Total phenolic content (mg GAE/g extract)	237.3 ± 8.5 ^a^
Total flavonoid content (mg QE/g extract)	180.5 ± 6.3 ^b^
Chemical content (µg/g dry weight)	
Kaempferol	115.0 ± 6.2 ^c^
Tannic acid	77.5 ± 3.0 ^d^
Quercetin	58.4 ± 1.6 ^e^
Isoquercitrin	25.5 ± 1.0 ^f^
Rutin	15.8 ± 0.9 ^g^
Apigenin	8.2 ± 0.4 ^h^
Gallic acid	5.6 ± 0.4 ^i^

NOTE: Data are expressed as mean ± SD (*n* = 3). Different superscript letters (a–i) indicate significant differences among mean values as determined by one-way analysis of variance (ANOVA) followed by Tukey’s post hoc test (*p* < 0.05).

## Data Availability

The data presented in this study are available on request from the corresponding author.

## Abbreviations

The following abbreviations are used in this manuscript:

°C	Degree Celsius
AO	Acridine Orange
ANOVA	Analysis of Variance
AP-1	Activator protein-1
ATCC	American Type Culture Collection
DMEM	Dulbecco’s Modified Eagle Medium
DMSO	Dimethyl sulfoxide
DNA	Deoxyribo Nucleic Acid
DPPH	2,2-Diphenyl-1-picrylhydrazyl
EB	Ethedium Bromide
EDTA	Ethylenediamine Tetra Acetic acid
ELISA	Enzyme-Linked Immunosorbent Assay
ERK	Extracellular signal-regulated kinase
FBS	Fetal Bovine Serum
g	Gram
GAE	Gallic Acid Equivalent
h	Hour
HPLC	High-Performance Liquid Chromatography
HT-29	Human Colorectal Adenocarcinoma cell
IC_50_	Half maximal inhibitory concentration
IL-1β	Interleukin-1 Beta
IL-6	InterLeukin-6
JNK	c-Jun N-terminal kinase
LPS	Lipopolysaccharides
M	Molar
MAPK	Mitogen-activated protein kinase
mg	Milligram
min	Minute
mL	Millilitre
MTT	3-(4,5-Dimethylthiazol-2-Yl)-2,5-Diphenyltetrazolium Bromide
NF-κB	Nuclear Factor κB
µM	Micro Molar
μg	Microgram
μL	Microlitre
nm	Nanometre
p38 MAPK	p38 Mitogen-activated protein kinase
PBS	phosphate-buffered saline
pg	Pico Gram
QE	Quercetin Equivalent
RAW-264.7	Murine macrophage cell line
RNase A	Ribonuclease-A
SD	Standard Deviation
TFC	Total Flavonoid Content
TNF-α	Tumor Necrosis Factor-Alpha
TPC	Total Phenolic Content
U/mL	Unit per Millilitre
UV	Ultra Violet
WBE	Winged Bean Pods Ethanolic Extract
